# Supplementary figures and images for: Design of nanobody targeting SARS-CoV-2 spike glycoprotein using CDR-grafting assisted by molecular simulation and machine learning
Source: PLoS Comput Biol. 2025 Apr 21;21(4):e1012921. doi: 10.1371/journal.pcbi.1012921 (PMC12068729; doi:10.1371/journal.pcbi.1012921)

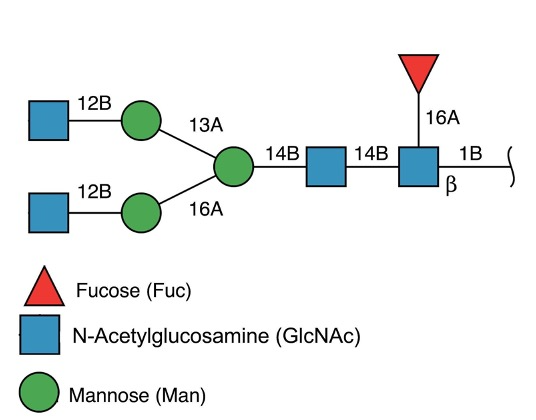

Supplement: S1 Fig — The N-glycan is composed of three components: N-Acetylgucosamine (depicted by a blue square), mannose (depicted by a green circle), and fucose (depicted by a red triangle). The α and β linkage types are represented by A and B, respectively. The numbering between the glycosidic linkages indicates the carbon involved in the bond, where the first number is the carbon number of the first monosaccharide and the second number is the carbon number of the second monosaccharide. (TIFF) [file pcbi.1012921.s002.tiff]

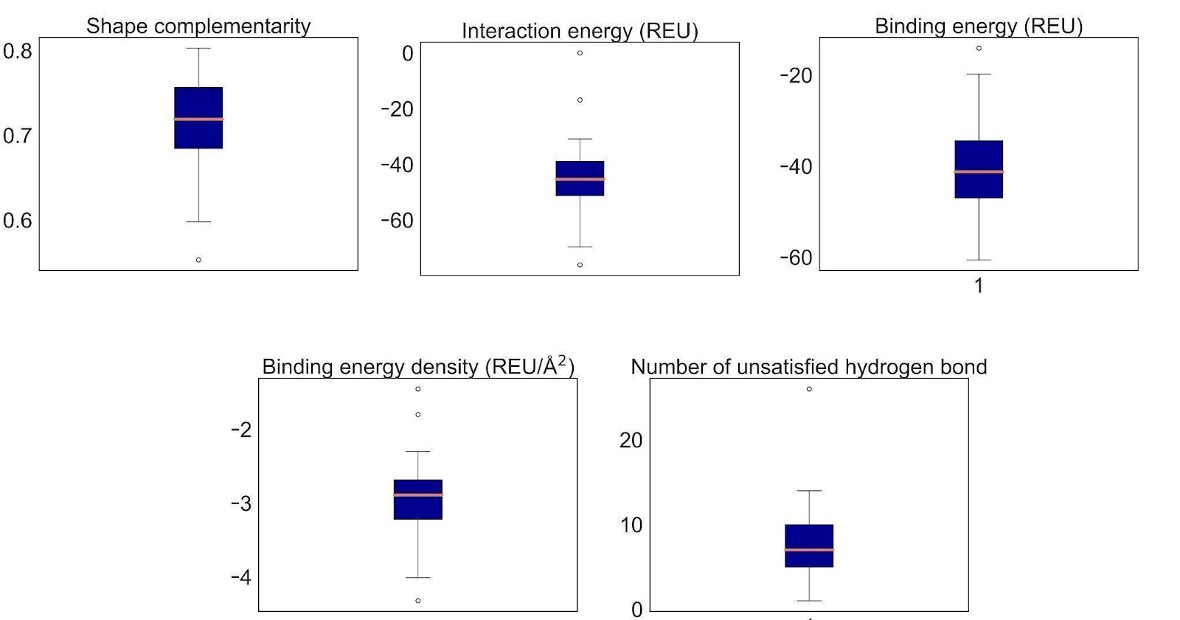

Supplement: S2 Fig — The interquartile range is shown as a solid blue box, where the top and bottom of the box denote the upper and lower quantile, respectively. The median is depicted as an orange line. Outliers are represented by circles. (TIFF) [file pcbi.1012921.s003.tiff]

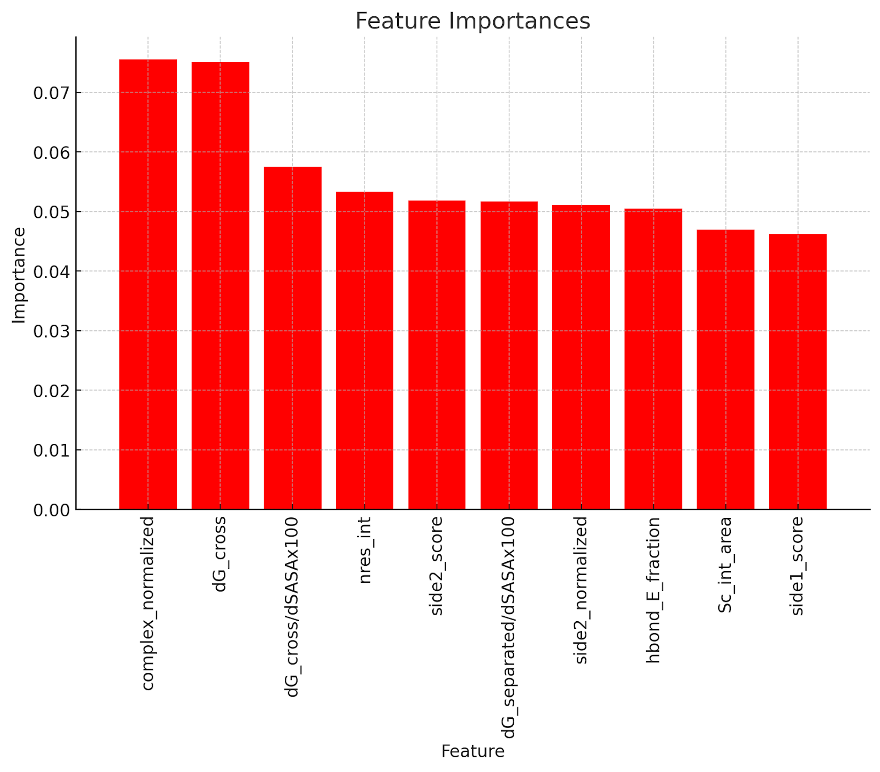

Supplement: S3 Fig — (TIFF) [file pcbi.1012921.s004.tiff]

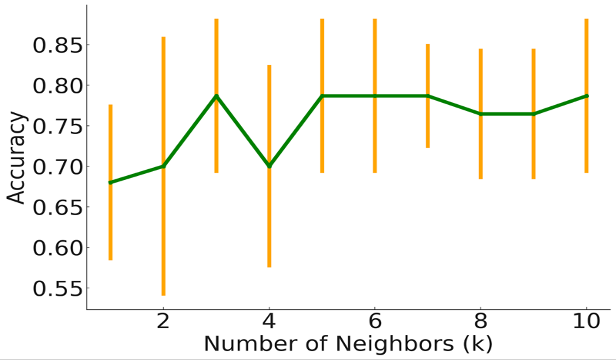

Supplement: S4 Fig — The blue line represents the mean accuracy, while the red bars indicate the standard deviation. Accuracy peaks at k=6, with a lower variability as compared to k=5. (TIFF) [file pcbi.1012921.s005.tiff]

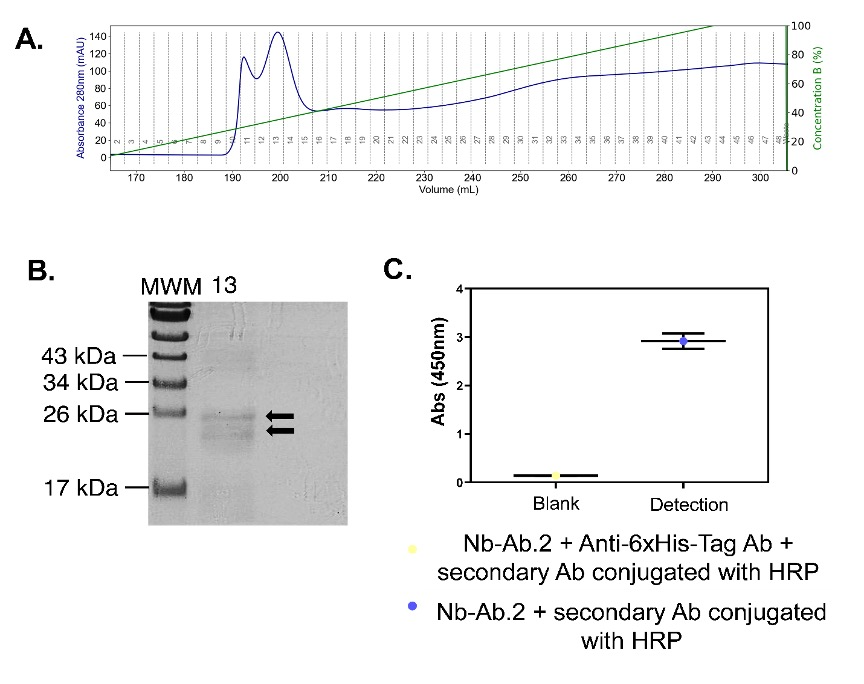

Supplement: S5 Fig — (A) Chromatogram from IMAC Purification of Nb Ab.2. The x-axis represents the elution volume (mL), while the left and right y-axes show the absorbance at 280 nm (A280) and the concentration of the elution buffer (%), respectively. (B) Fraction 13 was subjected to analysis using SDS-PAGE (17.5%). The nanobody Nb Ab.2 (19.5 kDa) migrated within the polyacrylamide gel with the expected mass, around 20 kDa. The occurrence of an additional band on the gel, positioned above the target band, is likely indicative of an incomplete removal of the pelB signal peptide from the N-terminal sequence of the nanobody [1]. The yield obtained was 0.5 mg per liter of bacterial culture. (C) Nb detection was made by using an in house His-Tag Protein ELISA assay. Plate was coated with the purified Nb during overnight. The next day, an anti-6xHis monoclonal antibody was added, followed by an HRP conjugated secondary antibody. We used the signal generated solely by the addition of the secondary antibody to the plate as our blank control. (TIFF) [file pcbi.1012921.s006.tiff]

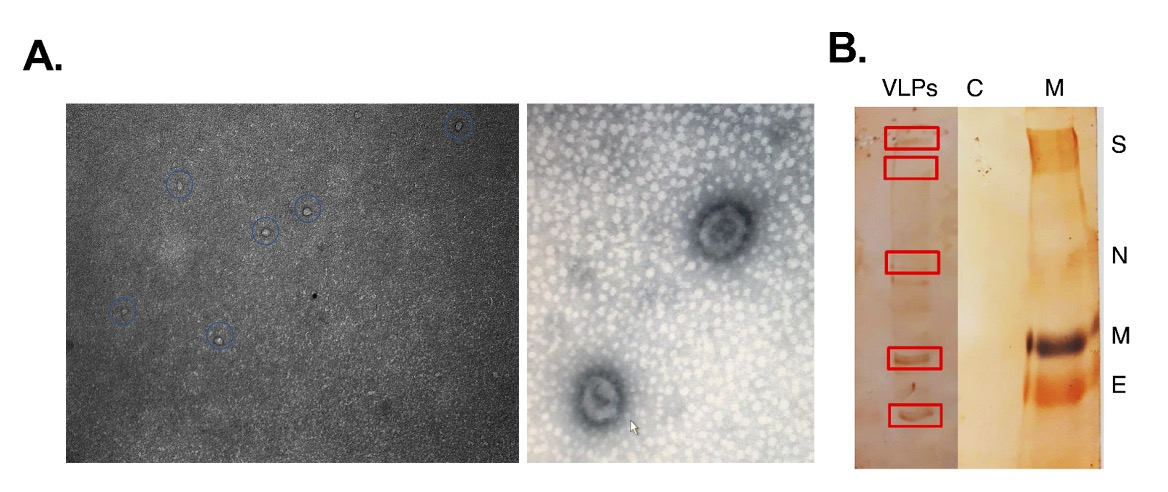

Supplement: S6 Fig — (A) Transmission electron microscopy (TEM) images of SARS-CoV-2 VLPs produced in Vero E6 cells 48 h after co-transfection with the plasmids encoding the virus structural proteins (M, S, E, and N) according to the 8:6:8:3 molar ratio. (B) Western-blot analysis of purified VLPs. Detection was performed using a pool of SARS-CoV-2-infected individuals. (TIFF) [file pcbi.1012921.s007.tiff]
