# Supplementary material for: Design of nanobody targeting SARS-CoV-2 spike glycoprotein using CDR-grafting assisted by molecular simulation and machine learning
Source: PLoS Comput Biol. 2025 Apr 21;21(4):e1012921. doi: 10.1371/journal.pcbi.1012921 (PMC12068729; doi:10.1371/journal.pcbi.1012921)
Supplement: Attachment — Submitted filename: Rebuttal_Letter-PlosCB.pdf [file pcbi.1012921.s011.pdf]

NEC OncoImmunity AS

Dr. Matheus Ferraz
Research Scientist
NEC OncoImmunity
www.oncoimmunity.com
Forskningsparken, Gaustadalléen
21, 0349, Oslo, Norway
matheus@oncoimmunity.com
ORCID:0000-0002-6958-3115

Mannheim, 11th January, 2025

Editorial Board

PLOS Computational Biology

Re: Rebuttal letter responding to each point raised by the editor and reviewer(s).

Title: Design of nanobody targeting SARS-CoV-2 spike glycoprotein using CDR-grafting assisted by molecular simulation and machine learning

Authors: Matheus Ferraz, Camilla Adan, Tayná Lima, Adriele Santos, Sérgio Paula, Rafael Dhalia, Gabriel Wallau, Rebecca C. Wade, Isabelle Viana, and Roberto Lins

Dear Editor(s) and reviewers,

We thank the opportunity to submit a revised version of the manuscript entitled "*Design of nanobody targeting SARS-CoV-2 spike glycoprotein using CDR-grafting assisted by molecular simulation and machine learning*" to *PLOS Computational Biology*.

We also thank for the time and effort that you all have allocated to provide valuable feedback on the manuscript. We have addressed all comments in the revised version, and corresponding changes (where applicable) are highlighted in **bold blue** in this letter and in the manuscript.

We would like to present a detailed, point-by-point response to the reviewer's comments below.

Reviewer #1

Comments to the author

> *This manuscript presents a grafting-based computational approach for designing nanobodies through combination of Rosetta-based modeling protocols, machine learning and molecular dynamics simulations. The*

approach was used to design nanobodies targeting the SARS-CoV-2 RBD, and the most promising one identified in-silico was experimentally tested to assess its stability and binding properties. The approach was found to be very effective in identifying a high-affinity binding nanobody without the need of high-throughput experimental screening (indeed only one nanobody was tested and was very active). This is an interesting manuscript and should be published after revising the following minor points.

Our response

We thank reviewer #1 for the supportive assessment.

Comment #1

Apparently, one limitation of this method lies in identifying a single CDR loop able to host all hotspot residues identified from alanine scanning on the original antibody. Here they managed to incorporate five hotspots in one loop of the synthetic library. But often hotspots coming from different CDRs could be hard to accommodate in a single loop, hence requiring more than one loop to accommodate enough hotspots to achieve high-affinity binding. I would like the authors to comment on how to address this.

Our response

We appreciate the reviewer's insightful observation regarding the challenge of incorporating multiple hotspot residues into a single CDR loop. Indeed, high-affinity binding may require accommodating hotspots across multiple CDR loops, especially when hotspots identified from alanine scanning are dispersed across distinct regions of the parental antibody. In our work, the employed methodology inherently addresses this limitation using the synthetic nanobodies' library by randomly grafting diverse CDRs onto the CabBCII-10 framework. Thus, this combinatorial diversity allows for multiple CDR loops to work synergistically, rather than relying on a single loop to incorporate all hotspot residues.

In scenarios where even this combinatorial strategy fails to accommodate all the identified hotspots simultaneously, this will be a limitation of the pipeline unless the protein designer chooses to reduce or prioritise a different subset of hotspots.

It is worth noting that this limitation is not specific to our method but is inherent to the *MotifGraft* approach, as has been thoroughly explored in prior publications.

To address this in the manuscript, we have added the following excerpt in the discussion section: "**In addition, high-affinity binding often requires the integration of multiple hotspot residues across different CDR loops. While the synthetic nanobody library inherently provides combinatorial diversity by randomly grafting CDRs onto the CabBCII-10 framework, there may still be instances where not all hotspots can be accommodated simultaneously. In such cases, the protein design process may require reducing or prioritizing a subset of hotspots. This limitation is inherent to the MotifGraft approach and should be considered when applying this methodology to highly complex binding interfaces.**"

Comment #2

> *500,000 designs were generated for a single grafted loop, but this sounds like too many sequences for just one starting backbone configuration and only enabling mutations in the CDR loops (likely many sequences are redundant). Please comment on this.*

Our response

We thank Reviewer #1 for raising this concern. To evaluate the redundancy in the generated sequences, we used CD-HIT with a sequence identity threshold of 95%, identifying approximately 120,000 redundant sequences. While a smaller library of generated sequences could suffice in principle, generating a larger library ensures a more thorough exploration of the sequence space. We agree that generating fewer sequences could be more computationally efficient, and we recommend that future users of this method adjust the library size based on their specific research needs.

To address this in the manuscript, we have added the following excerpt in the results section: "**To evaluate redundancy in the generated sequence library, we clustered sequences using CD-HIT at a 95% sequence identity threshold, identifying approximately 120,000 redundant sequences. Although smaller libraries may suffice for certain applications, generating a larger sequence library allows for thorough exploration of the sequence space. Future applications of this method could consider optimizing the library size to balance computational efficiency with design thoroughness.**"

Comment #3

> *At the beginning of the "Sequence filtering" section it is unclear how the random forest model was generated, which is confusing. I would add a reference to the Methods section, where it is described.*

Our response

We thank the reviewer for having pointed this out. After looking back at the scripts we observed we have used an extra trees model instead of random forest. We apologise for having overlooked it. In addition to correcting this in the main text, we have corrected it in the main text and added a short description in the SI, alongside the plot of the feature importance for each feature in the SI (S3 Figure).

Comment #4

> *For the AF3 prediction of the nanobody-RBD complex it would be more informative to provide the RMSD and pLDDT for the predicted CDR loops, as the nanobody framework is expected to have a high-quality prediction. Have the authors generated these predictions only using one seed? With extra seeds, as reported in AF3, the predictions should increase in quality. I would r recommend to check if the predictions improve in this way.*

Our response

Thank you for your valuable feedback. We appreciate your suggestion to provide the RMSD and pLDDT values for the predicted CDR loops, and we agree that this would offer more insight into the quality of the predictions. We have included these metrics in the revised version of the manuscript.

As suggested by the reviewer, we generated predictions using multiple seeds, and we observed that for the monomer Nb, the pLDDT values and the RMSD of the structures were very similar across different seeds. However, for the complex, changing the seed led to a decrease in prediction quality. This result is not entirely unexpected, as recent studies have shown that while AlphaFold3 offers improved predictions for antibody-antigen complexes compared to AlphaFold2, its accuracy remains limited. A recent comparative study of different structure prediction tools for antibody-antigen complexes confirmed these overall limitations in prediction accuracy (DOI: 10.1002/pro.5127).

Comment #5

> *MD simulations show that CDR3 of the designed nanobody has higher flexibility than the original loop of the parent nanobody (Fig. 4B). Is this related to differences in loop length? Also, only 1 replica has been done, which makes it difficult a fair comparison.*

Our response

Regarding the increased flexibility of the CDR3 loop in the designed nanobody, it is indeed likely influenced by the differences in chemical composition between the wild-type (WT) and designed nanobody. The modified sequence, as a result of the design process, could introduce changes in side-chain packing, hydrogen bonding, and other structural factors that contribute to different dynamic behaviour in the loop. Additionally, the loop dynamics of the WT nanobody is linked to its original function, which is binding to beta-lactamase. Since we re-designed the binding loops to make it optimal for a different binding partner, it's not unexpected the difference in dynamics due to sequence modifications.

We acknowledge the concern about the number of replicas used. While only one replica was performed, we have extended the production phase of the MD simulation to 1 μs to ensure a more thorough exploration of the conformational space. This longer simulation duration should help mitigate the limitations of a single replica and provide a more robust comparison of the flexibility between the WT and designed CDR3 loops. We have noticed that upon a longer simulated timescale, the CDR-1 also presents a higher flexibility for the designed Nb than that of the WT.

Comment #6

> *The same MD simulations should be extended or done in multiple replicas to really assess that the interface remains stable. Fig. 4D shows that the RMSD increases when reaching 100 ns, but 100 ns is a short time scale to really assess this (and in only 1 replica).*

Our response

We agree that a single 100 ns simulation, particularly with only one replica, may not be sufficient to thoroughly assess the stability of the interface over time. As you rightly pointed out, 100 ns is a relatively short timescale to capture the dynamics of the bound state, especially for systems with potential conformational changes. To address this, we have extended the production phase of the MD simulations to 1 μs for a more comprehensive analysis of the system's behaviour over a longer timescale.

We appreciate your insight and agree that longer simulations are necessary to draw more definitive conclusions about the system's behaviour.

Comment #7 and #8

> *Line 297-> F333 or F377?*
> *Line 298-> E57 or E56?*

Our response

Thank you for the corrections. We have changed it in the manuscript accordingly. We have highlighted the changes in bold blue in the manuscript.

Comment #9 and #10

> *Line 431 looks interrupted.*
>
> *Revise the phrasing of lines 445-447.*

Our response

Thank you for pointing this out. We have corrected this in the manuscript and now the paragraph is read as "**The experimentally derived k_D value of Nb Ab.2 binding to SARS-CoV-2 RBD corresponds to a binding free energy of -10.7 kcal/mol at room temperature (293.13 K). To compare this experimental value with structure-based computational models, we utilized an artificial neural network (ANN) model to predict the binding free energy for protein-protein complexes based on the designed structure. This ANN model, which has shown an average error of 1.6 kcal/mol for Nb-antigen complexes \cite{ferraz2023artificial}, predicted a binding free energy of -11.8 kcal/mol. The predicted value is within the method's margin of error, demonstrating consistency between the experimental data and the computational model based on the designed structure.**".

Reviewer #2

Comments to the author

> *The authors describe a pipeline to design nanobodies using a grafting strategy based on the Rosetta software suit. The strategy combines metadynamics simulations of the target in complex with a known antibody followed by Alanine scanning to determine the hotspot residues, which are*

then grafted onto a library of nanobodies (built by generating a pool of nanobodies by grafting distinct CDRs into a stable scaffold). Rosetta protocols are then used to introduce substitutions in the CDRs (except for the hotspots) and a costume-made ML-based function based on Rosetta metrics is used to filter the best candidates, which is followed by MD simulations. As a proof of concept, this strategy was used to target the SARS-CoV-2 spike protein receptor binding domain, yielding promising results. A selected candidate was evaluated experimentally, showing good binding affinity to the isolated RBD, which decreased when the evaluation was done in the presence of the whole spike protein. The work focuses on an important computational problem, namely the design of binders which contain loops at the interface. and presents a novel and potentially useful strategy. However, some points need to be addressed:

Our response

We thank reviewer #2 for the supportive assessment.

Comment #1

As a general comment the manuscript would highly benefit from including the neutralization assays and structural data to further show if and how the designed nanobody is able to neutralize the target.

Our response

We appreciate the reviewer's valuable suggestion regarding the inclusion of neutralization assays and structural data to strengthen the findings of our study. We fully agree that such experiments would provide significant insights into the neutralizing potential and structural basis of the designed nanobody's activity.

As mentioned in the manuscript (p. 19, line 660-665):

"Following the successful synthesis and characterization of a high-affinity Nb, future efforts will focus on assessing its therapeutic potential and understanding its molecular interactions. The next steps include evaluating the Nb's neutralizing capability against live virus *in vitro* and potentially extending these assessments to *in vivo* models to establish efficacy in more complex biological systems. Structural studies, such as X-ray crystallography, will elucidate the Nb's binding sites and conformation upon interaction."

These experiments are indeed crucial to subsequent pre-clinical studies, clinical trials, and eventually deploying a biopharmaceutical to the healthcare market; however, they fall outside the scope of the current study, which focuses primarily on the development and validation of the design pipeline. We anticipate that these efforts will be part of our future research, building upon the findings presented here.

Comment #2

> It is not clear if this strategy could be applied when no structures of antibodies bound to the epitope of interest are available. This limits its applicability scope and, in particular, will tend to focus on immuno-dominant epitopes, which is one of the biases that the authors are trying to avoid. This limitation should be discussed in the manuscript.

Our response

We thank Reviewer #2 for pointing this out. That is correct that our protocol relies on the availability of known interfaces, particularly when using the MotifGraft approach. In cases where crystal structures of antibody-antigen complexes are available, the design process can be more straightforward, as the structural information provides valuable guidance for grafting CDR loops.

However, in the absence of such structures, it is still possible to model the interface, although the accuracy of these models may vary depending on the quality of the predicted structure. In these cases, the design pipeline could potentially focus more on immuno-dominant epitopes, as you mentioned. We recognize that this could introduce biases, and this limitation should be considered when applying the method to targets without available structures.

To address this point, we have added the following sentence to the discussion section of the manuscript:
"**One limitation of the pipeline introduced in this work is that it relies on the availability of known antibody-antigen interface structures, as it uses the MotifGraft approach. While it is possible to model the interface using tools like deep learning-based methods (**10.1093/bioinformatics/btae583**,**10.3389/fimmu.2022.1053617**), the accuracy of the resulting designs may be lower compared to those based on experimentally determined structures. Additionally, in the absence of precise structural information, there may be a bias toward immuno-dominant epitopes.**"

Comment #3

> Nanobodies often contain disulfide bonds to stabilize them. Was this taken into account in this strategy?

Our response

Yes. We recognise the relevance of disulfide bond in Nbs structure, notably between CDR loops H1 and H3. Since these were present in the original Nanobody (PDB ID: 3DWT) we have kept it.

To address this in the manuscript we have added the following sentences in the methodological section: "**Disulfide bonds, particularly those between the CDR loops H1 and H3, are known to contribute to the stability of nanobodies. As these bonds were present in the original nanobody we built upon (PDB ID: 3DWT), they were preserved in the grafted designs to maintain structural integrity and stability.**"

Comment #4

Regarding metadynamics simulations, there are several questions that need to be clarified:

What was the rationale behind the choice of the CVs and why was the alpha-helical and b-sheet content of the CDRs chosen as a CV, since these regions are primarily composed by loops?

The relevant parameters used in the MetaD simulations, such as the height of the Gaussians and the frequency of deposition should be described.

Was convergence reached in the MetaD simulations?

Our response

The rationale behind the choice of collective variables (CVs) was based on their ability to capture the structural transitions and conformational preferences of the complementarity-determining regions (CDRs) during the metadynamics simulations. While it is true that CDRs are primarily loop regions, the secondary structure elements (e.g., α-helical or β-sheet content) were selected as CVs because they provide a coarse-grained representation of the conformational landscape, including potential structural rearrangements that may occur during antigen binding. We have used the same set of CVs as Löhr et al (10.3390/biom12050718), who used metadynamics to assess the dynamics of Nbs' loops. One of the main results Löhr et al show is that structural transitions in CDRs often involve transient β-sheet formation with the antigen or within the CDR itself, which can influence binding affinity. Capturing this behavior helps elucidate the relationship between structural dynamics and binding mechanisms.

We also thank the observation of this reviewer regarding the lack of the Gaussians parameters, and we agree that they should be presented. We apologise for having overlooked it. The parameters have been added to the methodological section.

Regarding the convergence of the metadynamics simulation, these were relatively short in this study (100 ns per replica, run in triplicate) to assure converged sampling. However, they were chosen for their ability to rapidly enhance sampling of conformational space, which was critical for our specific objectives. The goal of these simulations was not to achieve fully converged free energy landscapes but to efficiently sample diverse conformational states of the antibody CDR regions to identify key interactions and transitions relevant to binding specificity. We have observed that for this goal, previous studies have successfully utilized short metadynamics simulations to explore conformational transitions in antibody CDRs, even without full convergence, as referenced in our manuscript (10.3390/ijms23105419, 10.1002/pro.5176). These studies demonstrated that even short metadynamics runs could reveal biologically relevant structural features and transitions.

Comment #5

Regarding the PCA analysis performed on the MetaD and unbiased MD simulations, a detailed description of this analysis should be provided, since it is unclear which atoms were included and how the fitting was performed. Additionally it is unclear how the analysis was performed for the MetaD simulations, since a re-weighting would be necessary in this case to account for the correct Boltzmann distribution.

Our response

In the PCA analysis of the MetaD and cMD simulations, we included the backbone atoms of the loop H3, which were the areas of interest for our conformational analysis. The analysis was performed on the full trajectory, with no additional re-fitting, except for orientation to ensure consistency in comparison between simulations. We have added this information in the manuscript.

Regarding the re-weighting for the MetaD simulations, we understand that MetaD introduces a distribution different from the Boltzmann distribution, but since our goal was to compare the exploration of conformational space between MetaD and cMD, we did not apply re-weighting for the comparison between these two approaches. We acknowledge that a more rigorous re-weighting analysis may be needed in other contexts, but for the purpose of our comparison of sampling efficiency, we believe a direct comparison between the two approaches provides valuable insight into the enhanced sampling of MetaD.

Comment #6

In figure 2.0, how was the average and error calculated? Following in the previous question, was any re-weighting performed given that the frames come from MetaD simulations?

Our response

We have selected 10 frames from the metadynamics trajectory to capture representative conformations from diverse regions of the conformational landscape and these were used for alanine scanning. These structures were obtained from the most densely populated regions from the PCA space. Since alanine scanning evaluates energetic contributions for specific structures rather than thermodynamic distributions, reweighting was not applied for these analysis.

Comment #7

Regarding the nanobody library, what is the size and diversity of this library?

Our response

The final library comprises of nearly 80,000 synthetic Nbs with random CDRs on the CabBCII-10 framework. No redundant sequences at a 95% sequence identity threshold were observed.

To make this available to the reader, we have add the following sentence in the methodological section: "**The final library comprises of nearly 80,000 synthetic Nbs with random CDRs on the CabBCII-10 framework. No redundant sequences at a 95% sequence identity threshold were observed.**"

Comment #8

Concerning the ML-based function that was used to select the best candidates, how was the model selection and hyperparameter optimization performed? Were different ML-models tested?

Our response

We employed standard implementations from the scikit-learn library. The only hyperparameter requiring optimization was the number of neighbors (*k*) in the k-Nearest Neighbors (kNN) algorithm. To determine the optimal *k*, we tested a range of values and measured the accuracy of each configuration, as detailed in the Supplementary Information (SI).

While we tested various machine learning models during the development phase, only the combination of Linear Discriminant Analysis (LDA) with kNN yielded satisfactory results. For the sake of conciseness, we chose not to include the results of the less effective models in the manuscript.

Comment #9

Regarding the re-design of the interface residues, why did the authors choose to use the Rosetta package for this instead of methods like Protein MPNN which seem to generate very good results in this type of tasks?

We acknowledge that deep learning-based tools like ProteinMPNN have shown impressive results in protein design, including both backbone and sequence generation. However, at the time of our nanobody design work, these tools were not yet available.

Additionally, Li et al. (https://doi.org/10.1101/2024.12.16.628614) have demonstrated that ProteinMPNN, which was primarily trained on general protein datasets, struggled to effectively capture the unique sequence-structure relationships of antibodies/nanobodies. In particular, the model showed biases toward overrepresented residues and struggled to design variable regions, such as CDR3, which are critical for nanobody functionality.

We have included a comment on this on the discussion section.

Comment #10

It could be interesting to check if using the same CDRs without grafting the hotspots from the known antibody could also generate a good binder. This could serve as a control and as an assessment of what is the role the grafted hotsposts in binding.

Our response

We appreciate the suggestion and agree it is a relevant manner to show that the binding is a result of our design pipeline. We have modelled the system using the WT nanobody (PDB ID: 3WDT), which we built upon for our design, bound to the SARS-CoV-2 RBD by structural superimposition using our designed Nb:SARS-CoV-2 RBD as a reference. While our designed Nb bound to SARS-CoV-2 RBD displayed a binding energy of -37.27 REU (Rosetta energy units, computed from the InterfaceAnalyzer from Rosetta suit), the WT Nb bound to SARS-CoV-2 RBD displayed a positive binding energy of 13.82 REU, suggesting a much lower affinity. It's worth noting that for the modelling of the complexes, the same level of theory for relaxing the complexes' structure was employed.

We have included this extra information in the results section:
"**To ascertain that this affinity is a result of the design protocol, we compared the measured binding free energies using the Rosetta suite energy function. We have superimposed the structure of the WT Nb, which we built our design on, on the structure of the Nb Ab.2 and it displayed a positive binding energy of 13.82 REU, compared to the binding energy of -37.72 for the Nb Ab.2 - RBD complex. It's worth noting that for the modelling of the complexes, the same level of theory for relaxing the complexes' structure was employed, suggesting the WT Nb is not expected to binding to the SARS-CoV-2 RBD.**"

Comment #11

> *There is a problem with the text in page 13, line 431: there seems to be a mix of text from two different parts and this part of the text is incomprehensible.*

Our response

Thank you for pointing this out. We have corrected this in the manuscript and now the paragraph is read as "**The experimentally derived - k_D value of Nb Ab.2 binding to SARS-CoV-2 RBD corresponds to a binding free energy of -10.7 kcal/mol at room temperature (293.13 K). To compare this experimental value with structure-based computational models, we utilized an artificial neural network (ANN) model to predict the binding free energy for protein-protein complexes based on the designed structure. This ANN model, which has shown an average error of 1.6 kcal/mol for Nb-antigen complexes \cite{ferraz2023artificial}, predicted a binding free energy of -11.8 kcal/mol. This predicted value is within the method's error margin, demonstrating consistency between the experimental data and the computational model based on the designed structure**.".

Reviewer #3

Comments to the author

> *This study titled, "Design of nanobody targeting SARS-CoV-2 spike glycoprotein using CDR-grafting assisted by molecular simulation and machine learning", demonstrates a computational pipeline for designing nanobodies (Nbs) that*

bind with high affinity to the SARS-CoV-2 spike protein's receptor-binding domain (RBD). By integrating molecular simulations, metadynamics, machine learning, and motif grafting, the authors effectively design a nanobody (Nb Ab.2) with substantial experimental validation. Below are some suggestions and minor clarifications that could further enhance the manuscript's clarity and completeness.

General Comments:

- *The study is innovative and provides valuable insights into computational protein design. The design pipeline has the potential to make a significant impact on biotherapeutic development, particularly in addressing evolving viral threats.*
- *The integration of motif grafting, metadynamics, and machine learning is commendable and represents a holistic approach to nanobody design.*

Our response

We thank reviewer #1 for the supportive assessment.

Comment #1

The Materials and methods for the MD and metadynamics simulations is well-detailed. Adding information on the choice of the Nb CabBCII-10 scaffold for the grafting approach would provide useful context for readers.

Our response

The choice of using the CabBCII-10 framework as the scaffold for our design is based on the fact that our design pipeline centers around the grafting of CDRs. We needed a framework that could reliably accommodate diverse CDR loops while maintaining overall structural stability. The CabBCII-10 scaffold, proposed by Vincke et al. (2009), is known for its versatility and robustness in this regard. It was specifically designed to serve as a universal framework capable of supporting various CDRs, which makes it an ideal starting point for our grafting approach. In our case, this scaffold allows for the introduction of a wide range of CDR loop combinations without compromising the stability of the nanobody.

This has been addressed in the original manuscript in the introduction and discussion sections.

Comment #2

Line 263-284: The rationale for using metadynamics to enhance sampling space is well-argued. Additional quantitative metrics on the efficiency of metadynamics in identifying conformational states not captured by conventional MD would strengthen the results.

Our response

To address the comment, we applied the GROMOS clustering method to the conformational ensembles generated by both conventional MD and metadynamics simulations. Using an RMSD

cutoff of 2 Å, we identified 3 clusters for conventional MD and 7 clusters for metadynamics, demonstrating that metadynamics samples a broader range of conformational states. Additionally, we calculated the explained variance for the first principal component (PC1) and found that metadynamics captured 68% of the variance, compared to 57% for conventional MD. This indicates that metadynamics not only enhances conformational diversity but also better captures the dominant motions within the system.

We have added these information in the discussion section.

Comment #3

> *Line 305: Only heavy-chain residues were grafted due to their higher binding contribution. Given the role of light-chain interactions in some antibody-antigen bindings, could the authors discuss if future designs might incorporate light-chain residues to increase binding stability?*

Our response

Thank you for your insightful comment. In our work, only the heavy-chain residues were grafted due to their higher contribution to the binding affinity as demonstrated by the alanine scanning results. However, the grafting of residues can accommodate residues from both light and heavy chain. The limitation of this are geometric constraints which would hinder the identification of proper conformations in our library. However, this is a limitation inherent to the MotifGraft approach.

To address this point, we have added the following sentence to the manuscript:
"**While only heavy-chain residues were grafted in this study due to their higher contribution to the binding affinity, incorporating light-chain residues is also viable to enhance binding stability, depending on the specific antigen and desired interactions**."

Comment #4

> *Line 315-320: The sequence filtering process via machine learning was insightful. It may benefit from a brief clarification regarding the choice of the 10 Rosetta interface metric. Were these chosen solely for classification importance, or did they also have biophysical significance?*

Our response

The 10 Rosetta interface metrics were selected based on their classification importance in the context of identifying and distinguishing binding strength between high and low. These metrics were chosen to provide relevant features for the classification task, rather than for their direct biophysical significance. While some of these metrics do have biophysical relevance, as has been discussed in the manuscript (e..g, hydrogen bond energy, binding free energy per solvent-accessible surface area, and the normalized energy of the antigen), the primary criterion for their inclusion was their ability to improve the performance of the machine learning model in distinguishing high- or low-affinity binding interfaces.

Comment #5

Line 407-417: The CD spectrum confirms secondary structure integrity in Nb Ab.2. A more detailed breakdown of the CD data concerning the secondary structure content would be helpful to clarify specific folding patterns in the redesigned nanobody.

Our response

To better clarify the folding patterns, we have added the following to the results section:

"**The quantitative analysis of secondary structure content was performed using BeStSel method, which provides precise quantification of beta-sheet content, distinguishing between parallel and anti-parallel beta-sheets. This is essential for Nbs, whose stability relies heavily on well-defined beta-sheet structures. The Nb is composed mostly of antiparallel β-sheets (47.3%). Other structural elements account for 33% of the Nb's composition and include the CDR domains. Additionally, BeStSel also classified as a fragment of immunoglobulin G, formed by sandwich-like β-sheets. The secondary structure content of Nb-BCII10 was determined using the PDB entry 3WDT as input in the BeStSel software. Accordingly, 3WDT is composed of 40.2% of antiparallel β-sheets and 47.4% of other elements. The proportion of parallel β-sheets calculated for Nb Ab.2 (7.3%) was slightly higher than that predicted for thse reference structure (4.3%) and is likely attributable to the CD signal observed at 230 nm (https://doi.org/10.1016/j.ab.2019.113491).**"

Comment #6

Line 429-459: The reduced affinity of Nb Ab.2 for the full spike protein compared to the isolated RBD is noted, attributed to conformational differences. Have the authors considered strategies such as affinity maturation or further CDR optimization to improve binding affinity in this context?

Our response

Thank you for your valuable observation. The reduced affinity of Nb Ab.2 for the full spike protein compared to the isolated RBD is not unexpected, as our computational design pipeline was based on the structure of the isolated RBD. The design process targeted the RBD to optimize interactions specifically with that domain, and it is likely that the full spike protein's conformational complexity contributed to the observed reduction in affinity.

To improve the binding affinity of the nanobody to the full spike protein, we hypothesize that using the full spike structure as input in the design pipeline would allow for more accurate optimization of interactions across the entire protein. This could help account for the conformational differences and better tailor the nanobody to the spike's full structure.

To incorporate this suggestion, we have added the following sentence to the discussion section of the manuscript: "**To improve the binding affinity to the full spike protein, future design iterations could consider using the full spike structure as the input, allowing for a more comprehensive optimization of the nanobody's interactions to the specific conformation of the spike protein.**"

In addition to the comments above, Reviewer #2 suggested that the code for the ML-based filtering function should be provided. This has been added to the accompanying Jupyter-notebook section on GitHub.

Once again, we appreciate the opportunity to submit a revised version of the manuscript, and hope the undertaken changes and comments would be satisfactorily.

We look forward to hearing from you,

Sincerely,

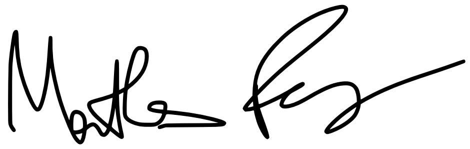

Matheus Ferraz, on behalf of the authors
Research scientist, NEC OncoImmunity AS
