# Supplementary material for: Design of nanobody targeting SARS-CoV-2 spike glycoprotein using CDR-grafting assisted by molecular simulation and machine learning
Source: PLoS Comput Biol. 2025 Apr 21;21(4):e1012921. doi: 10.1371/journal.pcbi.1012921 (PMC12068729; doi:10.1371/journal.pcbi.1012921)
Supplement: Attachment — Submitted filename: plos_review.docx [file pcbi.1012921.s010.docx]

**Comments to the Authors**

This study titled, “*Design of nanobody targeting SARS-CoV-2 spike glycoprotein using CDR-grafting assisted by molecular simulation and machine learning*”, demonstrates a computational pipeline for designing nanobodies (Nbs) that bind with high affinity to the SARS-CoV-2 spike protein's receptor-binding domain (RBD). By integrating molecular simulations, metadynamics, machine learning, and motif grafting, the authors effectively design a nanobody (Nb Ab.2) with substantial experimental validation. Below are some suggestions and minor clarifications that could further enhance the manuscript's clarity and completeness.

General Comments:

- The study is innovative and provides valuable insights into computational protein design. The design pipeline has the potential to make a significant impact on biotherapeutic development, particularly in addressing evolving viral threats.
- The integration of motif grafting, metadynamics, and machine learning is commendable and represents a holistic approach to nanobody design.

Specific Comments and Questions:

1. The Materials and methods for the MD and metadynamics simulations is well-detailed. Adding information on the choice of the Nb CabBCII-10 scaffold for the grafting approach would provide useful context for readers.
2. Line 263-284: The rationale for using metadynamics to enhance sampling space is well-argued. Additional quantitative metrics on the efficiency of metadynamics in identifying conformational states not captured by conventional MD would strengthen the results.
3. Line 305: Only heavy-chain residues were grafted due to their higher binding contribution. Given the role of light-chain interactions in some antibody-antigen bindings, could the authors discuss if future designs might incorporate light-chain residues to increase binding stability?
4. Line 315-320: The sequence filtering process via machine learning was insightful. It may benefit from a brief clarification regarding the choice of the 10 Rosetta interface metric. Were these chosen solely for classification importance, or did they also have biophysical significance?
5. Line 407-417: The CD spectrum confirms secondary structure integrity in Nb Ab.2. A more detailed breakdown of the CD data concerning the secondary structure content would be helpful to clarify specific folding patterns in the redesigned nanobody.
6. Line 429-459: The reduced affinity of Nb Ab.2 for the full spike protein compared to the isolated RBD is noted, attributed to conformational differences. Have the authors considered strategies such as affinity maturation or further CDR optimization to improve binding affinity in this context?

Overall, this study makes a significant contribution to computational nanobody design and is likely to attract interest from researchers in protein engineering and therapeutic development. Thank you for sharing this important and innovative work.
